# The mouse *Char10* locus regulates severity of pyruvate kinase deficiency and susceptibility to malaria

**DOI:** 10.1371/journal.pone.0177818

**Published:** 2017-05-18

**Authors:** Aurélie Laroque, Gundula Min-Oo, Mifong Tam, Prem Ponka, Mary M. Stevenson, Philippe Gros

**Affiliations:** 1 Biochemistry Department, McGill University, Montreal, Quebec, Canada; 2 Department of Microbiology and Immunology, McGill University, Montreal, Quebec, Canada; 3 Lady Davis Institute for Medical Research, Jewish General Hospital, Montreal, Quebec, Canada; 4 Physiology Department, McGill University, Montreal, Quebec, Canada; Institut national de la santé et de la recherche médicale - Institut Cochin, FRANCE

## Abstract

Pyruvate kinase (PKLR) deficiency protects mice and humans against blood-stage malaria. Although mouse strain AcB62 carries a malaria-protective *Pklr*^*I90N*^ genetic mutation, it is phenotypically susceptible to blood stage malaria induced by infection with *Plasmodium chabaudi* AS, suggesting a genetic modifier of the *Pklr*^*I90N*^ protective effect. Linkage analysis in a F2 cross between AcB62 (*Pklr*^*I90N*^) and another PK deficient strain CBA/Pk (*Pklr*^*G338D*^) maps this modifier (designated *Char10*) to chromosome 9 (LOD = 10.8, 95% Bayesian CI = 50.7–75Mb). To study the mechanistic basis of the *Char10* effect, we generated an incipient congenic line (Char10C) that harbors the *Char10* chromosome 9 segment from AcB62 fixed on the genetic background of CBA/Pk. The *Char10* effect is shown to be highly penetrant as the Char10C line recapitulates the AcB62 phenotype, displaying high parasitemia following *P*. *chabaudi* infection, compared to CBA/Pk. Char10C mice also display a reduction in anemia phenotypes associated with the *Pklr*^*G338D*^ mutation including decreased splenomegaly, decreased circulating reticulocytes, increased density of mature erythrocytes, increased hematocrit, as well as decreased iron overload in kidney and liver and decreased serum iron. Erythroid lineage analyses indicate that the number of total TER119^+^ cells as well as the numbers of the different CD71^+^/CD44^+^ erythroblast sub-populations were all found to be lower in Char10C spleen compared to CBA/Pk. Char10C mice also displayed lower number of CFU-E per spleen compared to CBA/Pk. Taken together, these results indicate that the *Char10* locus modulates the severity of pyruvate kinase deficiency by regulating erythroid responses in the presence of PK-deficiency associated haemolytic anemia.

## Introduction

Malaria is one of the clearest and best studied examples of an infectious disease which intensity and outcome are strongly influenced by genetic factors of the host[1,2]. This includes loss-of-function variants of erythrocyte-specific proteins (so-called hemoglobinopathies), which cause severe and life-threatening disease in the homozygous state, but which heterozygosity exerts a protective effect against blood-stage or cerebral malaria. This is the case of hemoglobin variants associated with sickle cell anemia (HbS), or α and β thalassemias, HbC, HbE, as well as G6PD deficiency, Duffy negativity, and Melanesian ovalocytosis (Band 3 protein)[3–5]. In addition, in the major blood groups, A and B have been found to be associated with a greater risk of severe malaria in some studies, suggesting a protective role of the O blood group[6]. Finally, genome wide studies, and numerous studies with candidate genes have identified variants in genes involved in host inflammatory (TNFα, IFNγ, NOS2A, LTA, IRF1) and immune responses (HLA, FCGR2A) that are associated with malaria severity and/or outcomes[1,2]. Finally, these complex genetic effects are further modulated by poorly identified environmental factors, including density of the insect vector populations, virulence determinants of the *Plasmodium* parasites (including resistance to anti-malarial drugs), poor nutritional status of the host (anemia), and co-infection with other accidental or endemic bacterial or helminth pathogens[7,8].

Genetic studies in mouse models of blood stage (*Plasmodium chabaudi AS*) and cerebral malaria (*Plasmodium berghei ANKA*) have proven valuable to identify genetic determinants of susceptibility or resistance to infection[9–24]. The relevance of these candidate genes to pathogenesis and host response to the human malarial parasite (*Plasmodium falciparum*) can then be tested in human population studies. A striking example is the *Char4* locus, one of the 11 mapped *Char* loci (*Cha**baudi* resistance), which control susceptibility (parasitemia at the peak of infection, overall survival) to *P*. *chabaudi AS* in inbred, recombinant inbred, and recombinant congenic strains of mice. The *Char 4* locus was identified as protective against blood stage malaria in strains AcB55 and AcB61. These two strains are derived from the highly susceptible A/J parental strain and harbor susceptibility alleles at the major *Char1* and *Char2* loci[15,16]. *Char4*-determined malaria-resistance in AcB55 and AcB61 was shown to be caused by homozygosity for a loss-of-function mutation at the *Pklr* gene (*Pklr*^*I90N*^) that encodes the erythrocyte specific pyruvate kinase[25]. Pyruvate kinase catalyses the last step of glycolysis, converting phosphoenolpyruvate to pyruvate with the generation of one molecule of ATP. PK-deficiency in AcB55/AcB61 causes hemolytic anemia, extramedullary erythropoiesis, reticulocytosis, and protection against infection (low parasitemia, survival) [25,26]. The malaria-protective phenotype of PK-defciency was found to be recapitulated in an independent mutant variant (*Pklr*^*G338D*^) from the CBA/N-Pk^slc^ strain (referred to as CBA/Pk later in the text)[27].

Pyruvate kinase deficiency is the most frequent abnormality of the glycolytic pathway and is the second most common cause of non-spherocytic hereditary hemolytic anemia in humans. Importantly, studies of human erythrocytes from 3 PK-deficient patients infected *ex vivo* with *P*. *falciparum* showed that homozygosity for PK-deficient alleles causes a dramatic reduction in parasite replication and increased phagocytosis of parasitized erythrocytes. *P*. *falciparum* infected erythrocytes from heterozygotes were also more avidly phagocytozed than control infected erythrocytes[28]. Finally, re-sequencing the *PKLR* gene in human populations (21 ethnic groups, 6 geographical clusters) from current or ancestral areas of malaria endemicity (Africa, South-East Asia) identified a rich genetic diversity at human *PKLR*, and have suggested that the gene may be under selection[29,30].

AcB62 is another recombinant congenic strain that carries the malaria-protective *Pklr*^*I90N*^ mutation. Yet, AcB62 is susceptible to *P*. *chabaudi* with high peak parasitemia (~60%) compared to AcB55/61 (~35%) and to C57BL/6J resistant controls (~35%), suggesting the presence of a genetic modifier of the *Pklr*^*I90N*^ malaria-protective effect in this strain[19]. A genome scan in PK-deficient [AcB62 X CBA/Pk]F2 mice infected with *P*. *chabaudi* was conducted using peak parasitemia as a quantitative measure of susceptibility. These F2 mice are informative for mapping this modifier, as they are fixed for PK deficiency, although the CBA/Pk allele (*Pklr*^*G338D*^) is more severe than the *Pklr*^*I90N*^ allele of AcB62[27]. Linkage analysis identified a major locus on chr 9 controlling parasitemia in infected animals, that we designated *Char10* (LOD = 10.8; 95% confidence interval 51.3Mb-68.3Mb). There was an additional effect on chr 3 (LOD = 3.8; near *Pklr*), due to *Pklr* mutant alleles of different severity segregating in this cross[19,27].Hence the *Char10* locus is a modifier of the malaria-protective effective effect of PK-deficiency.

In this study, we have studied the mechanism by which the *Char10* locus modulates the penetrance and expressivity of pyruvate kinase deficiency in the AcB62 mouse strain.

## Materials and methods

### Mice

**Fig 1:**
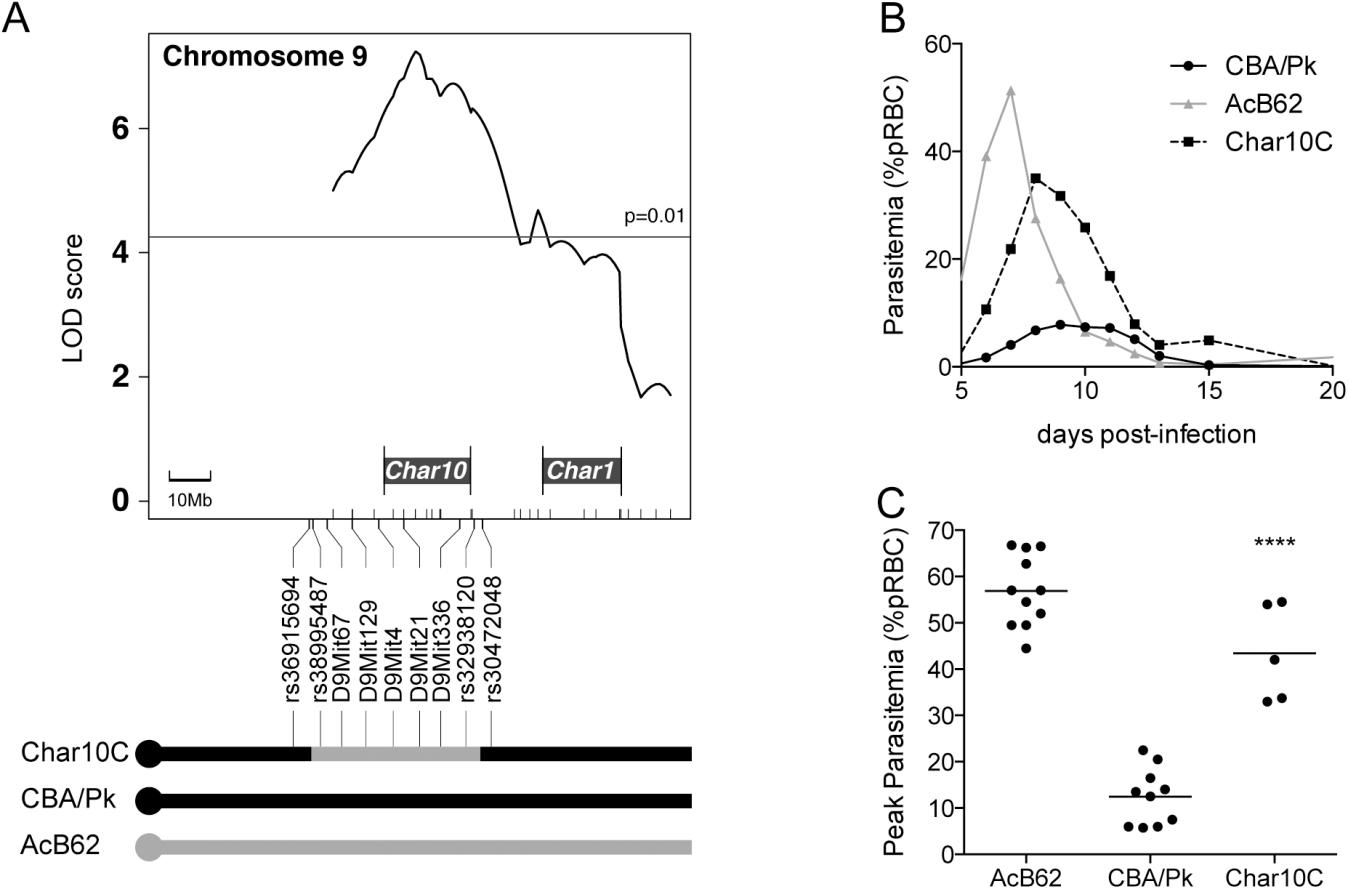
Construction and phenotyping of the Char10C incipient congenic line. (A) Physical delineation of the chromosome 9 (*Char10*) segment from the AcB62 strain backcrossed onto the genetic background of the CBA/Pk strain. The LOD trace was adapted from Min-Oo *et al*. 2010[19]. The schematic representation of the recombinant chromosome 9 is shown below for the Char10C incipient congenic line and for parental CBA/Pk and AcB62. The positions of informative markers used for genotyping and the length of the chromosome 9 transferred are given in megabases and are drawn to scale. (B) Course of *Plasmodium chabaudi* AS infection (blood parasitemia) in CBA/Pk, AcB62 and Char10C mice over a period of 20 days. Five to eleven animals per group were profiled. (C) Peak parasitemia levels are shown for strains CBA/Pk, AcB62 and Char10C. Each dot represents one mouse. Statistical significance (two-tailed Student’s *t*-test; compared to CBA/Pk) is indicated by stars: ****P<0.0001.

A/J and C57BL/6J mice were purchased from the Jackson Laboratory (Bar Harbor, ME, USA). The AcB recombinant congenic strain AcB62 was generated according to a breeding scheme of Demant and Hart[31] and has been described previously[15]. CBA/Pk mice were provided by the Japan SLC Animal Facility (Mr H Asai). Char10C incipient congenic mice were generated from CBA/Pk (background strain) and AcB62 (donor strain) using marker-assisted backcrossing (Fig 1A); Markers *D9Mit25*, *D9Mit4*, *D9Mit208*, *D9Mit336*, *D9Mit341*, *D9Mit51* were used to monitor presence of the AcB62 alleles at the *Char10* locus in the Char10C line. All strains were maintained under pathogen-free conditions in the animal facility of McGill University and handled according to the guidelines and regulations of the Canadian Council on Animal Care. Mice experimentation protocol was approved by the McGill Facility Animal Care Committee (P. Gros, Principal Investigator; protocol number: 5287), and includes procedures to minimize distress and improve welfare. All mice were gender-matched in each individual experiments to control for gender effects.

### Parasite and infection

A lactate dehydrogenase virus-free isolate of *P*. *chabaudi chabaudi* AS, originally obtained from Dr Walliker (University of Edinburgh), was maintained by weekly passage in A/J mice by intraperitoneal infection with 10^6^ parasitized red blood cells (pRBCs) suspended in 1mL of pyrogen-free saline. Experimental mice were infected intravenously with 10^5^ pRBCs and the percentage of pRBCs was determined daily on thin blood smears stained with Diff-Quick (Dade Behring, Newark, DE, USA) on days 4 to 21 after infection. Survival from infection was monitored twice daily, everyday of the experiment, and moribund animals were sacrificed. A monitoring log, placed in the mouse room, was signed by research personnel following each check. We did not experience any unexpected deaths. Humane endpoints were used during all of our animal survival studies, and in accordance with the McGill University FACC (Facility Animal Care Committee). At any time, if any of our experimental mice exhibited signs of distress such as weight loss exceeding more than 20% body weight, a body condition score (BCS) < 2, moderate to severe dehydration, decreased mobility, loss of appetite, ruffled fur with loss of grooming behavior, lethargy and or hunched posture, these mice were immediately euthanized (carbon dioxide exposure) following anesthesia with isoflurane.

### Hematological parameters

Fresh whole blood was collected in heparinized tubes by cardiac puncture from nine to ten animals per strain. Red blood cell counts, haemoglobin, haematocrit, and Mean Corpuscular Volume (MCV) were determined by the Diagnostic and Research Support Service of the Animal Resources Centre of McGill University. Reticulocytes counts were performed on methylene-blue-stained thin blood smears; four fields of 100 cells were counted per mouse.

### Histology

Spleen, liver and kidney were obtained from CBA/Pk, Char10C and C57BL/6 mice, fixed overnight in 10% formalin (neutral buffered), dehydrated in ethanol/xylene, and embedded in paraffin wax. Histology sections were cut on a microtome at 4 μm and fixed to glass slides. Sections were de-paraffinized in xylene, rehydrated in a series of ethanol baths, and then stained for iron using Perl’s Prussian blue solution, dehydrated in ethanol/xylene, and mounted with Acrytol (Leica Biosystems, Canada).

### Iron-related and erythropoietin (EPO) measurements

Iron biochemical determination in spleen, liver and kidney was performed according to the method described by Torrance and Bothwell[32]. Briefly, iron quantification was done by acid digestion of tissue samples at 65°C for 20h, followed by colorimetric measurement of the absorbance of the iron-bathophenanthroline complex at 535nm. Quantitative measurements of plasma iron, total iron binding capacity (TIBC) and transferrin saturation were performed at the Molecular Diagnostic laboratory of the Jewish General Hospital, Montreal, Canada. Plasma EPO levels were quantified using a sandwich enzyme-linked immunosorbent assay (ELISA) (R&D Systems, Minneapolis, MN, USA) according to the manufacturer’s guidelines.

### Flow cytometry

Spleens and bone marrow were harvested. Single cell suspensions were prepared in PBS supplemented with 2% FBS and 2mM EDTA and filtered through a 40μm cell strainer (BD Biosciences, Mississauga, ON, Canada) to remove cell aggregates. Single cell suspensions were counted manually and viability determined by trypan blue exclusion was always >95%. Cells (1x10^6^) were then incubated with 0.5 μg of anti-CD16/CD32 monoclonal antibodies (eBioscience Inc, San Diego, CA, USA) prior to staining with the following fluorescence-conjugated antibodies purchased from eBioscience: anti-mouse TER119-PE (clone TER119), anti-mouse CD71-FITC (clone R17217) and anti-mouse CD44-APC (clone IM7). Non-viable cells were excluded using 7-AAD viability staining solution (eBioscience Inc, San Diego, CA, USA). Cells were acquired using BD FACSCanto II (BD Biosciences, Mississauga, ON, Canada) and data were analysed using FlowJo version 9.3.3.

### Erythroid colony-forming unit (CFU-E) assays

Single cell suspensions were prepared from CBA/Pk, Char10C and C57BL/6J spleen and bone marrow in IMDM (Life Technologies, ON, Canada) supplemented with 10% FBS and 100U/mL penicillin/streptomycin (Thermo Scientific, UT, USA). Spleen or bone marrow were harvested from 3 mice per group and pooled. Cells were plated into 1% methylcellulose medium supplemented with 15% FBS, 1% BSA, 10 μg/mL insulin and 200 μg/mL transferrin (M3234, Stem Cell Technologies, BC, Canada). Various concentrations (0–200 mU/mL) of erythropoietin (R&D Systems, Minneapolis, MN, USA) as well as 2 mM of L-glutamine (Life Technologies, ON, Canada) were added to the medium. Cells were plated in 35 mm dishes (Sarstedt, Montreal, Canada) at a density of 4x10^4^ cells/mL for spleen and 2x10^4^ cells/mL for bone marrow. CFU-E were scored after 2.5 days of culture in a 5% CO_2_ humidified incubator kept at 37°C.

### Biotinylation of RBCs

*In vivo* biotinylation of erythrocytes was performed as we have described[27]. Briefly, the entire RBC population was biotinylated at *t* = 0 following i.v. injection of 0.1 ml of sulfo-NHS-biotin (Pierce, Rockford, IL) at a concentration of 50 mg/mL and the frequency of biotinylated RBCs in the blood was determined by flow cytometry. Five microliters of blood was taken from tail vein and diluted in 1 mL of PBS. Cells were counted and approximately 1 × 10^8^ RBCs were stained with 3 μg PE-streptavidin (BD Biosciences, Mississauga, ON) in 0.2 ml PBS for 30 min at 4°C. After washing with PBS, the stained cells were analyzed by FACSCalibur using CellQuestPro software. The frequency of the biotinylated RBCs in the blood was followed at regular intervals during a period of 30 days.

Adoptive transfer of biotinylated RBC was performed as described previously[33]. Briefly, blood from CBA/N donor was collected and washed three time in PBS supplemented with 0.1% glucose and was then incubated for 15 min in 0.1 mg/mL biotin-NHS at room temperature. It was then washed once with PBS supplemented with 0.1% glucose and 100mM glycine, and a second time with PBS alone. 1.9x10^9^ RBCs in 0.3 mL were injected i.v in each recipient mouse. The frequency of biotinylated RBCs was analysed during a period of 50 days.

### Statistical analysis

Statistical significance between groups was tested by an unpaired, two-tailed Student *t*-test. The data were analysed using GraphPad Prism 6.0 statistical software. *P*-values <0.05 were considered significant. *P<0.05; **P<0.01; ***P<0.001; ****P<0.0001.

## Results

### Creation and characterization of a *Char10* incipient congenic mouse line

*Char10* was previously shown to regulate parasitemia levels at the peak of infection in pyruvate kinase deficient [AcB62xCBA/Pk] F2 mice, and was mapped to the proximal portion of chromosome 9[19]. This cross segregates two *Pklr* mutations, the *Pklr*^*I90N*^ allele from AcB62 and the more severe *Pklr*^*G338D*^ mutation from CBA/Pk[27]. This complicates the study of the mechanism of action of the *Char10* locus and its effect on the malaria-protective phenotypes imparted by PK-deficiency in these F2 mice. To initiate the identification of the cellular and biochemical pathways underlying the *Char10* effect, we used a marker-assisted strategy to construct a incipient congenic line in which the chromosome 9 portion overlapping the *Char10* locus from AcB62 (donor strain) was transferred by serial backcrossing (4 generations) to the genetic background of CBA/Pk. The resulting line, designated Char10C, was genotyped for informative markers on chromosome 9, and was found to be homozygote for an AcB62 chromosome 9 segment spanning from 35.21Mb to 74.88Mb (Fig 1A). The Char10C line is also homozygote for CBA/Pk derived *Pklr*^*G338D*^ mutant alleles on chromosome 3, as expected. In all subsequent experiments, comparative studies in the Char10C and CBA/Pk lines were performed to gain insight into the function of *Char10*.

To validate the modulating effect of *Char10* on PK-deficiency associated malaria phenotype, we infected AcB62, CBA/Pk and Char10C mice with *P*. *chabaudi chabaudi* AS and followed blood parasitemia over time. Char10C mice were found to be susceptible to *P*. *chabaudi chabaudi* AS infection, with peak parasitemia levels (43.5%) resembling those seen in the parental AcB62 (57%), and clearly distinct from those detected in the CBA/Pk mutant (12.48%) (Fig 1B and 1C). These results demonstrate that the *Char10* locus (AcB62-derived alleles) is sufficient to suppress the malaria-protective effect of PK-deficiency (*Pklr*^*G338D*^) in CBA/Pk. Furthermore, the *Char10* effect is fully penetrant as the incipient congenic line recapitulates the phenotype of the AcB62 parental line (peak parasitemia).

### *Char10* modulates the extent of the PK deficiency-associated anemia

**Fig 2:**
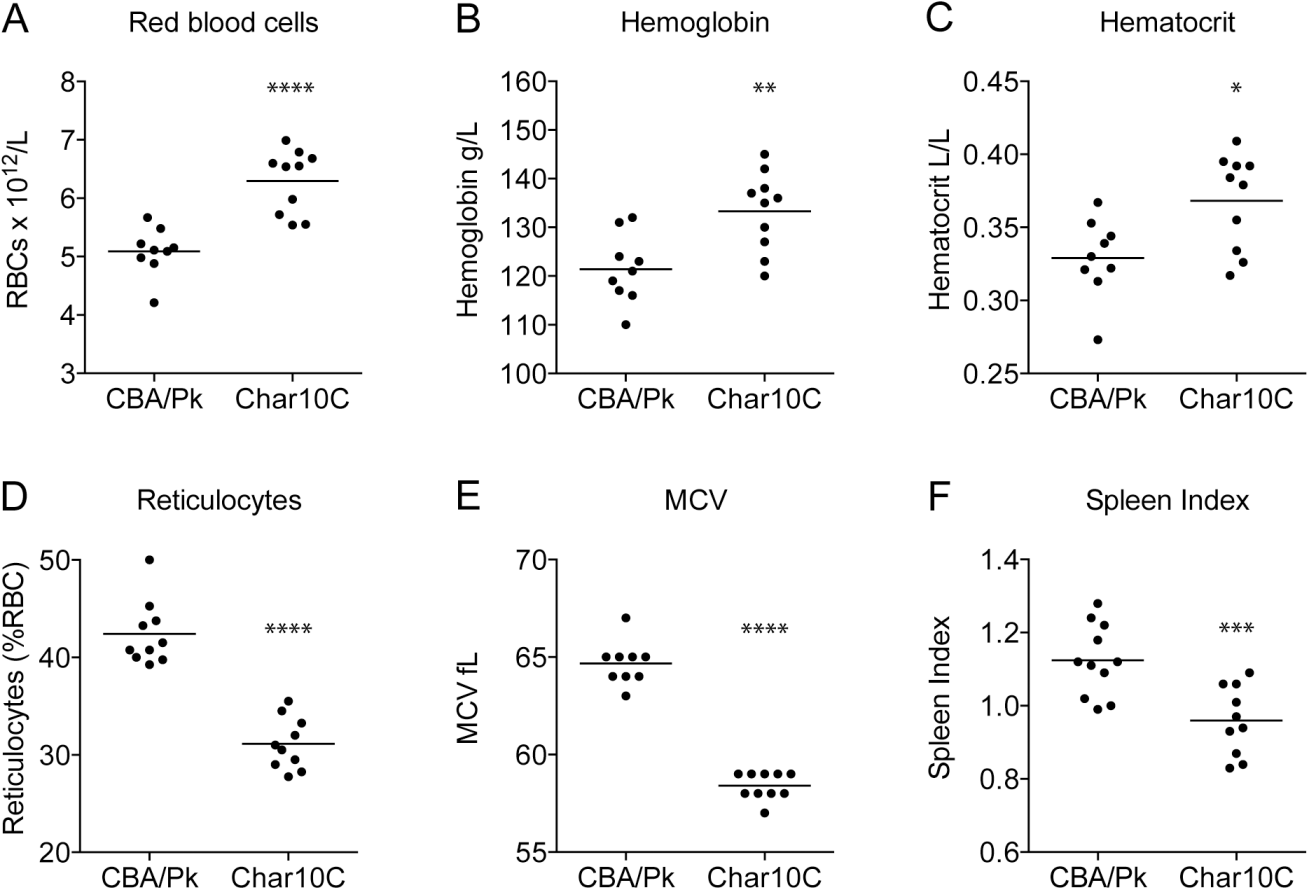
Comparative hematological profile of the Char10C incipient congenic line. Hematological parameters were obtained in naïve adult mice (>8 weeks of age). Abbreviations: RBCs, red blood cells; MCV, mean corpuscular volume. Each dot represents one mouse. Statistical significance (two-tailed Student’s *t*-test; compared to CBA/Pk) is indicated by stars: *P<0.05; **P<0.01; ***P<0.001; ****P<0.0001.

Protection against blood-stage malaria in PK-deficient AcB55, AcB61 and CBA/Pk has been linked to anemia, and to altered properties of PK-deficient erythrocytes, such as high reticulocytosis and shortened half-life or mature red cells in peripheral blood. In addition, we have observed a quantitative correlation between intensity of anemia phenotypes (reticulocytes) and resistance to malaria (peak parasitemia) in individual [AcB62xCBA/Pk] F2 and [AcB55 x A] F2 mice[25–27]. Therefore, we investigated the effect of the *Char10* locus on the anemia-associated phenotypes characteristic of PK-deficiency, initially by comparing haematological profiles of Char10C and CBA/Pk (Fig 2). Char10C mice are significantly less anemic than CBA/Pk mice. They show higher numbers of erythrocytes (6.3 vs 5.1 x 10^12^/L), higher hematocrit (0.37 vs. 0.33L/L), and lower numbers of circulating reticulocytes (31.1% vs. 42.4%). Char10C mice also have a lower mean corpuscular volume (MCV) of total RBCs, which is probably due to the lower proportion of reticulocytes. Finally, and although tissue section identified intense expansion of the spleen red pulp and associated secondary erythropoiesis in both mouse strains, Char10C mice show a highly significant reduction in splenomegaly when compared to CBA/Pk (Fig 2F). This initial haematological profiling suggests that the *Char10* locus modulates intensity of PK-deficiency in CBA/Pk.

### *Char10* modulates the extent of PK-deficiency-induced iron overload in peripheral tissues

**Fig 3:**
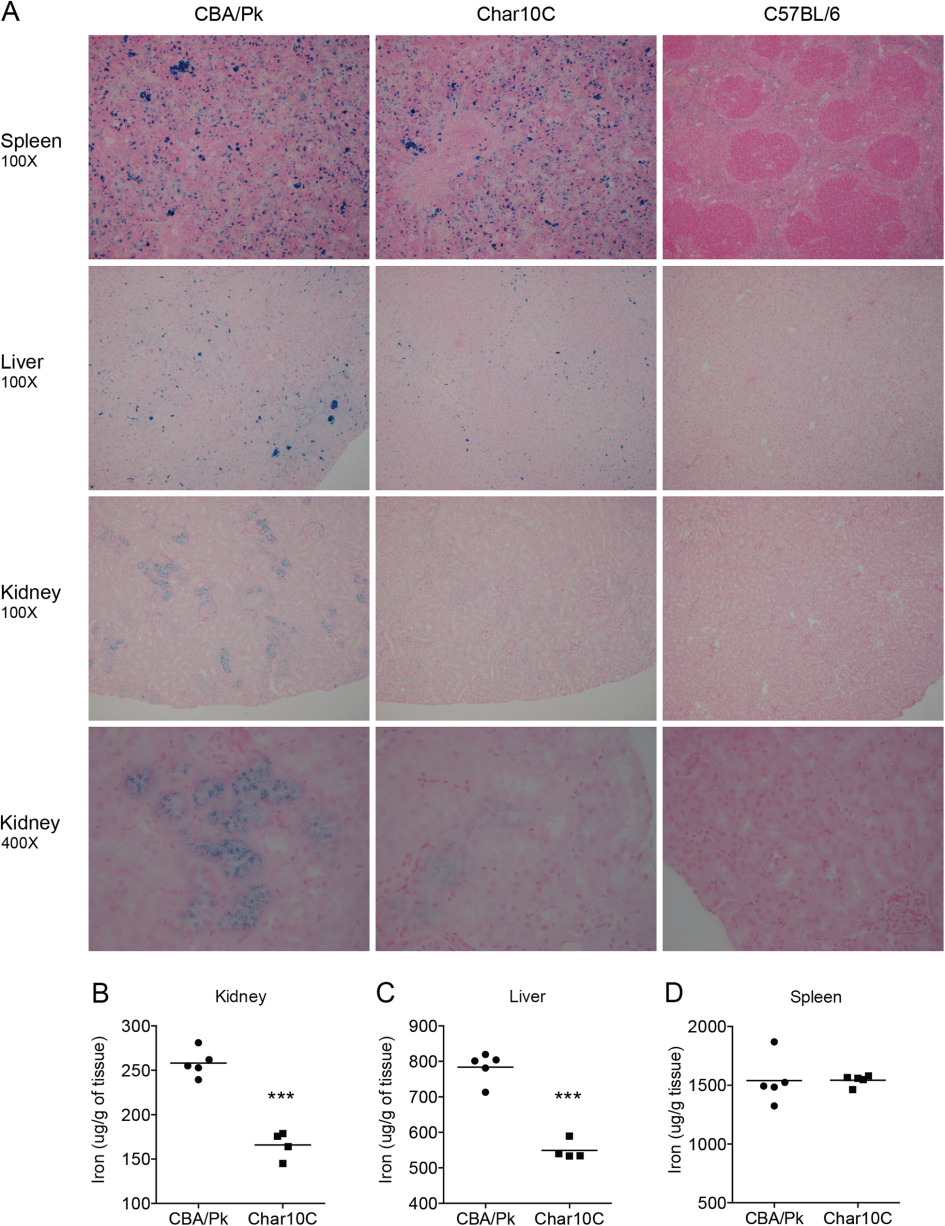
Iron determination in peripheral tissues. Spleen, liver and kidney of CBA/Pk, Char10C and C57BL/6 mice were harvested and used for histochemical and biochemical determination of iron content. (A) Histochemical staining of spleen, liver and kidney sections stained with Perl’s Prussian blue. (B) Iron biochemical quantification in spleen, liver and kidney extracts. Each dot represents one mouse. Statistical significance (two-tailed Student’s *t*-test; compared to CBA/Pk) is indicated by stars: ***P<0.001.

**Fig 4:**
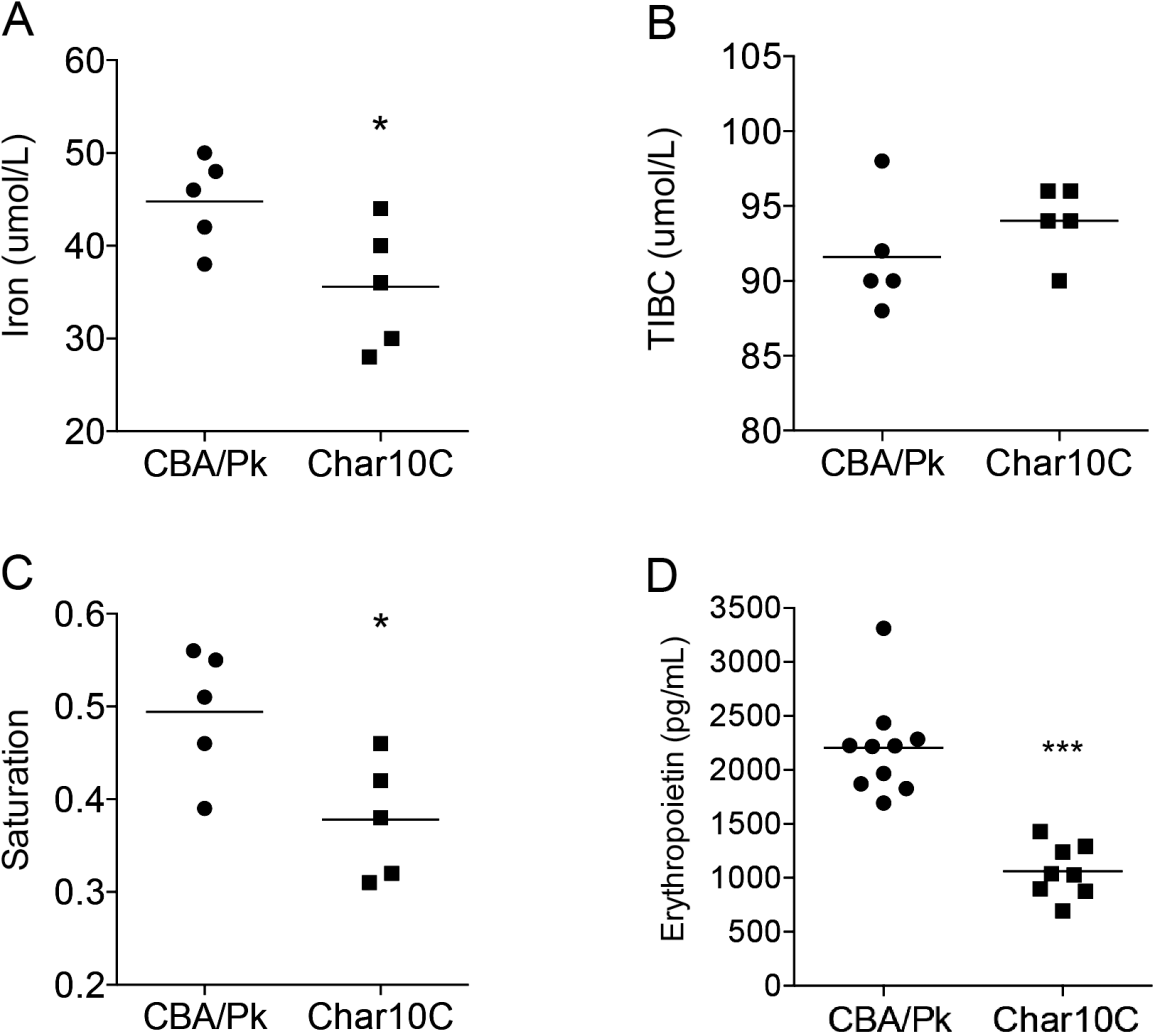
Iron-related measurements in plasma. Comparative analyses of plasma from CBA/Pk vs Char10C mice. (A) Iron total concentration. (B) Total iron-binding capacity. (C) Transferrin saturation. (D) Erythropoietin concentration. Each dot represents one mouse. Statistical significance (two-tailed Student’s *t*-test; compared to CBA/Pk) is indicated by stars: *P<0.05; ***P<0.001.

Hemolytic anemia in conditions such as PK-deficiency are often accompanied by iron overload in peripheral tissues[34,35]. We investigated whether *Char10* had an effect on iron metabolism at such sites. Staining of tissue sections with Perls’ Prussian blue showed reduced iron in liver and kidney of Char10C mice (but not spleen), when compared to CBA/Pk (Fig 3A). In liver, iron deposits appeared mostly in mononuclear phagocytes (Kupffer cells), while in kidney, iron deposits appeared mostly at the apex of epithelial cells of proximal tubules. Further quantification of iron in organ extracts confirmed results of histochemistry and identified significantly lower iron stores in liver and kidney in Char10C mice compared to CBA/Pk (Fig 3B, 3C and 3D). Char10C mice also showed lower plasma iron levels and lower plasma transferrin saturation than in CBA/Pk mice (Fig 4A and 4C), while total iron binding capacity (TIBC) was similar in both strains (Fig 4B). Erythropoietin (EPO) is critical for erythrocyte production in response to anemia[36]. We determined that plasma levels of EPO in Char10C mice were about half that of CBA/Pk (Fig 4D). Taken together, iron and EPO measurements in tissue and plasma provide complementary information that *Char10* modulates the severity of haemolytic anemia caused by Pklr deficiency.

### Effect of *Char10* regulation on metabolism of PK-deficient erythrocytes

**Fig 5:**
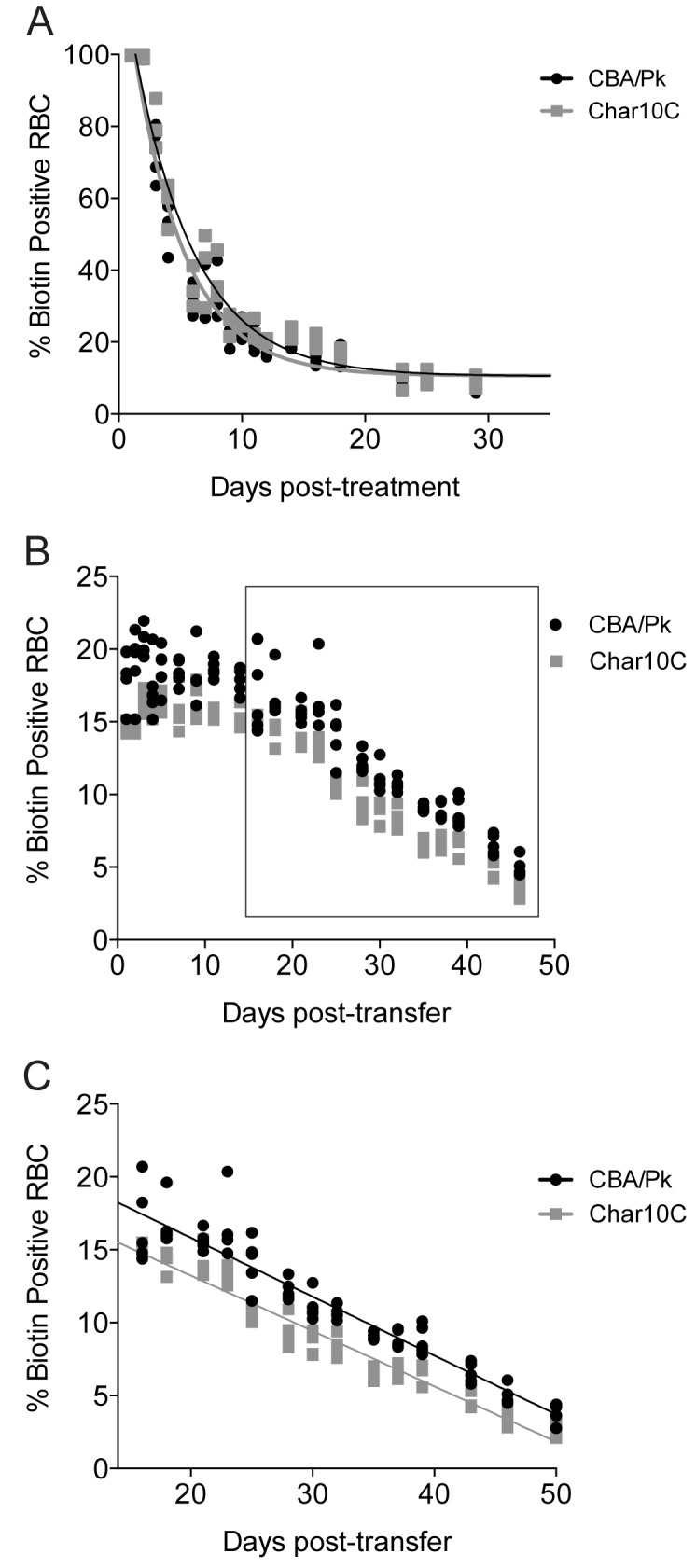
Effect of Char10 on turnover of biotinylated erythrocytes. (A) Turnover of *in vivo* biotinylated erythrocytes from CBA/Pk and Char10C mice, as determined by flow cytometry. (B) Turnover of *ex vivo* biotinylated control CBA/N erythrocytes following adoptive transfer into CBA/Pk and into Char10C mice, and as measured by flow cytometry over a period of 50 days. (C) Enlargement of the linear portion of the graph shown in panel B.

Results so far indicate that *Char10* alleles impact the severity of anemia phenotypes (erythrocyte and reticulocyte numbers, plasma iron and tissue iron stores) in PK-deficient CBA/Pk and Char10C mice. This could reflect differential fragility of *Pklr*^*G338D*^ deficient erythrocytes produced in CBA/Pk vs. Char10C mice, perhaps resulting in haemolytic signals of different intensities. Alternatively, this could suggest *Char10*-regulated hematopoietic responses of different intensities in response to the same *Pklr*^*G338D*^ associated haemolytic insult. To distinguish between these two possibilities, we determined the half-life of *Pklr*^*G338D*^ deficient erythrocytes produced in CBA/Pk vs. Char10C mice using an *in vivo* biotinlylation assay we have previously described[27]. In this protocol, biotin is injected intravenously, and the disappearance of biotin-labeled erythrocytes is monitored daily by FACS, and erythrocytes half-life is determined (Fig 5A). These experiments showed an identical half-life of ~5 days for erythrocytes produced in both mouse lines. The half-life of erythrocytes can also be influenced by the pace at which they are removed from the circulation. Hence, we used a modification of this protocol to assess the capacity of the reticulo-endothelial system (macrophages) of CBA/Pk and Char10C to eliminate normal erythrocytes (from wild type PK-sufficient CBA/N) labelled *ex vivo* with biotin and injected back in both mouse strains (Fig 4B and 4C). These experiments showed that CBA/Pk and Char10C mice could eliminate normal RBCs at the same rate. Hence the *Char10* locus does not modulate erythrocytes half-life either directly, or through differential rates of removal by the reticuloendothelial system.

### *Char10* regulation of erythropoiesis in PK-deficient mice

**Fig 6:**
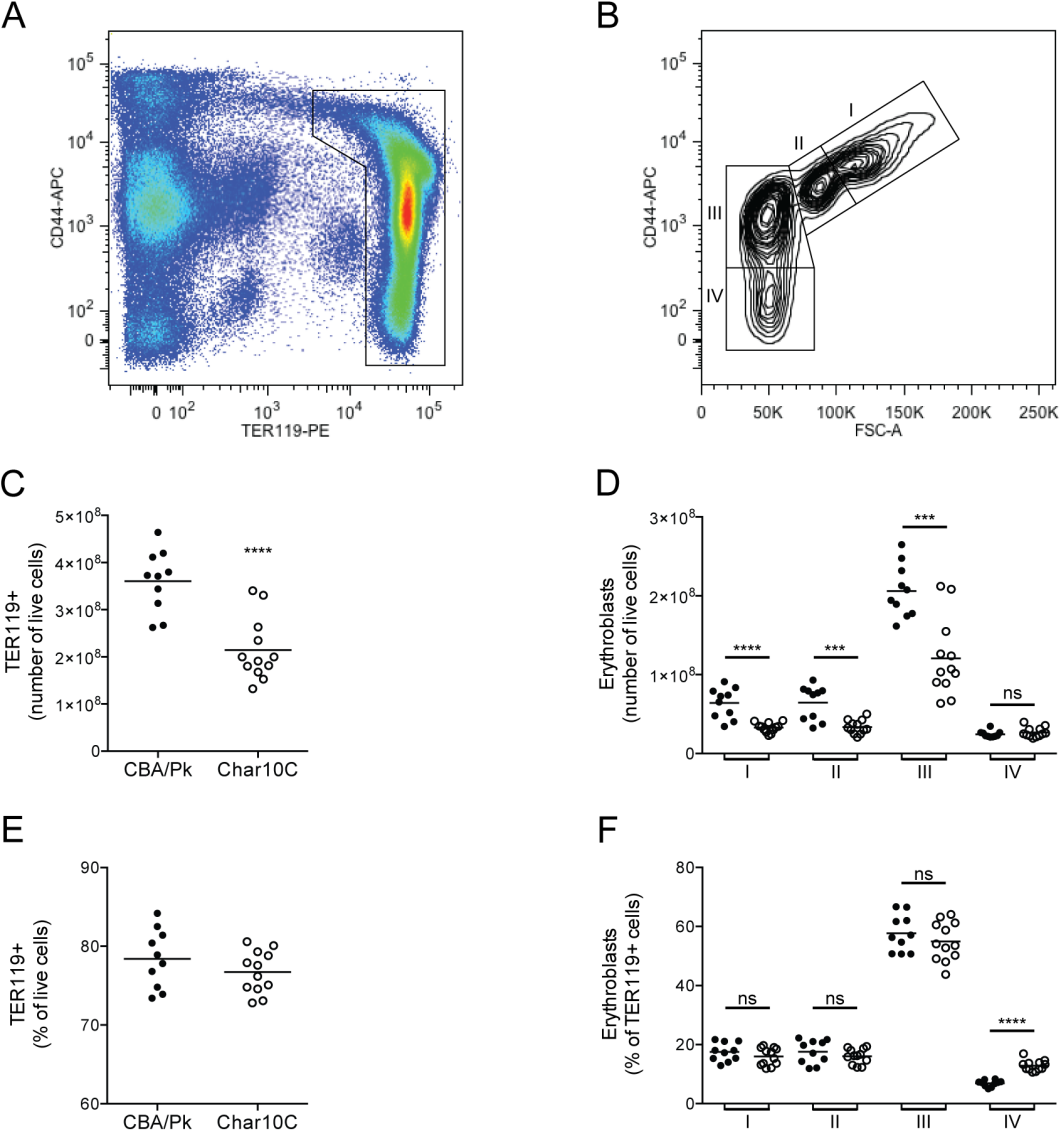
Flow cytometric analysis of the spleen erythroid compartment. (A) Single spleen cells suspensions from CBA/Pk and Char10C mice were labelled with antibodies against TER119 and CD44. (B) Distribution of CD44+ populations versus FSC of TER119+ gated cells was used to identify 4 sub-populations of erythroblasts (I to IV) as described [38]. The total number of Ter119+ erythroblasts (C) and of the 4 sub-populations of erythroblasts (D) is shown. (F) Erythroblasts expressed as percentage of TER119+ cells. Each dot represents one mouse, and all experiments were done in triplicate. Statistical significance (two-tailed Student’s *t*-test; compared to CBA/Pk) is indicated by stars: ***P<0.001; ****P<0.0001.

We investigated a possible effect of *Char10* alleles on erythroid response to haemolytic anemia. For this, we used FACS analysis to distinguish and to quantify the different populations of maturing erythroblasts from spleen, the major site of ertyhropoiesis in PK-deficient mice. In these studies, TER119 was used as a general marker of the erythroid lineage and CD71 (transferrin receptor; data not shown) and CD44 (hyaluronic acid receptor) as maturation markers for the erythroid lineage. The expression of CD71 and CD44 in TER119^+^ erythroblasts decreases with maturation, and when combined with analysis of cell size, this previously described strategy[37,38] permits the identification of four erythroblast maturation stages (Fig 6A and 6B). The number of total TER119^+^ cells, as well as the numbers of the different erythroblast sub-populations (labelled I to IV) were all found to be lower in Char10C spleen compared to CBA/Pk controls (Fig 6C and 6D). This is consistent with the higher splenomegaly of CBA/Pk compared to Char10C, and strongly suggests increased erythropoiesis in the former CBA/Pk compared to Char10C. Interestingly, we noted that the relative percentage of TER119^+^ cells in the spleen, or the respective percentage of each erythroblast sub-populations was similar in CBA/Pk and Char10C (Fig 6E and 6F).

**Fig 7:**
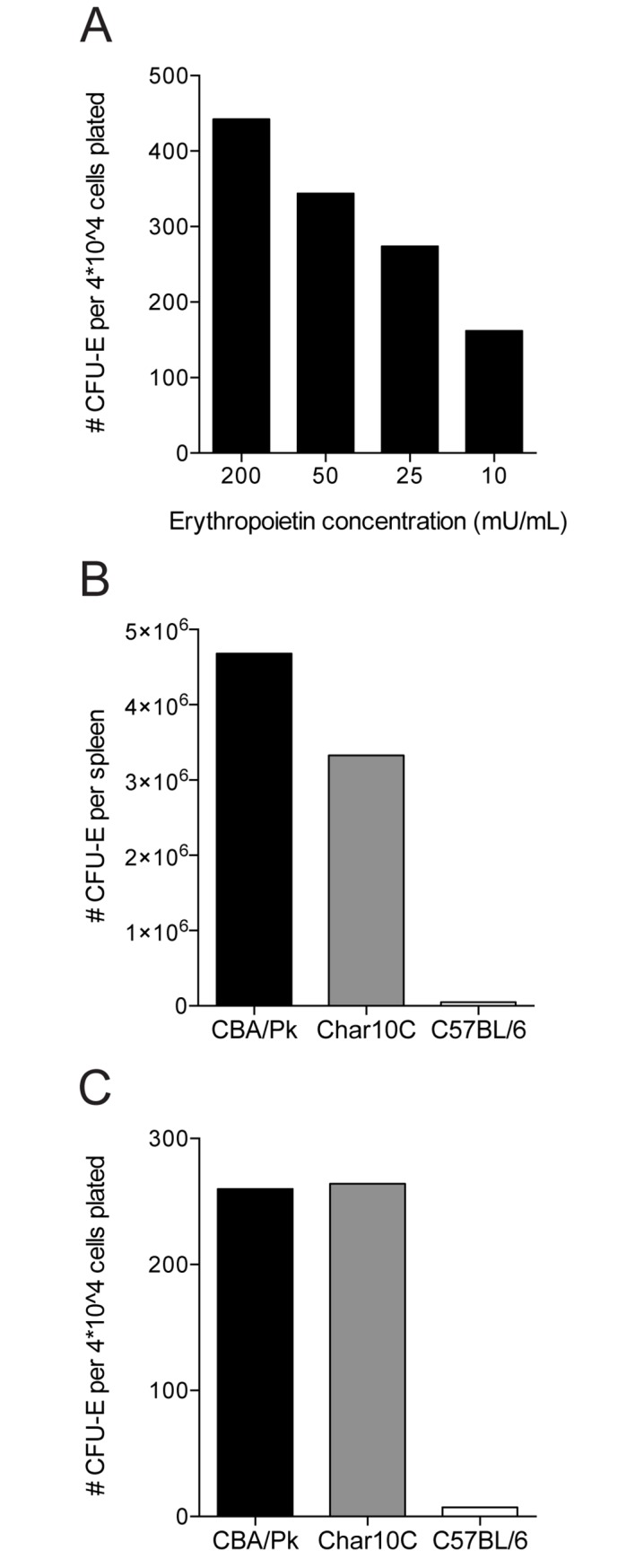
Enumeration of CFU-E progenitors in spleens. Spleens of three male CBA/Pk, Char10C and C57BL/6 mice were pooled for analysis. (A) Erythropoietin (EPO) titration was performed on CBA/Pk spleens to determine sub-optimal EPO concentrations required to stimulate CFU-E colony formation. Histograms represent the mean of two technical replicates. Erythropoietin titration. (B) Total number of CFU-E per spleen determined following culture in EPO at 10mU/mL (C) Proportion of CFU-E colonies per 4x10^4^ cells plated at 10mU/mL EPO.

To determine whether the *Char10* effect on both the splenic TER119^+^ erythroid cell populations and circulating reticulocytes may be linked to altered maturation of early erythroid progenitors, we measured the potential of splenic cells from both strains to form CFU-E *in vitro* in response to EPO. Dose-response experiments suggested that 10U/mL EPO was sub-optimal and non-saturating, and was selected for our assay conditions (Fig 7A). The total number of CFU-E per spleen was found to be lower in Char10C than in CBA/Pk (Fig 7B). This result suggests more robust erythropoiesis in CBA/Pk than in Char10C mice, in agreement with the higher number of TER119^+^ erythroid precursors detected in the spleen of these mice by FACS analysis (Fig 6). On the other hand, the relative frequency of CFU-E formed per 4x10^4^ cells plated was found to be the same in the two strains (Fig 7C).

Taken together, these results strongly suggest that the *Char10* locus differentially modulates erythroid response to the same PK-deficiency haemolytic stimulus. The *Char10* effect appears to be at the level of global erythroid response as opposed to the intrinsic ability of erythroid precursors to fully differentiate into erythrocytes.

## Discussion

Erythrocyte senescence in haemolytic anemia such as that encountered in pyruvate kinase deficiency and during *Plasmodium* infection is associated with decreased ATP levels, heme deposition, and oxidative damage which cause decreased deformability, retention in red pulp splenic sinuses and phagocytosis by residents macrophages[39–41]. Hemolytic anemia is associated with compensatory erythropoiesis in the spleen (also in liver and bone marrow), with noticeable splenomegaly, increased numbers of TER119^+^ erythroblasts in these organs, and of circulating reticulocytes. This chronic compensatory erythropoiesis is associated with increased intestinal iron uptake and ultimately, iron overload in peripheral tissues. We showed previously that in mice and humans, PKLR-deficiency is associated with increased resistance to malaria[25,26,28]. Resistance may be accounted for by a) diminished replication of *Plasmodium* parasites in the unfavourable environment of reduced ATP content of PK-deficient erythrocytes[42], or b) reduced half-life and increased phagocytosis of PK-deficient RBCs, including *Plasmodium* infected ones[28], or c) a combination of both. The malaria-protective effect of PK-deficiency is phenotypically similar to that of inactivation of other erythrocyte proteins (G6PD, Band-3, DARC), including hemoglobin (sickle cell anemia, thalassemias)[3].

In three recombinant congenic strains that share common ancestry (AcB55, AcB61, AcB62), we identified a severe loss of function in Pklr (*Pklr*^*I90N*^) that accounts for the *Char4* locus. This locus is associated with protection against *P*. *chabaudi* induced blood-stage malaria in AcB55 and AcB61[25]. In AcB61, the protective effect of *Pklr*^*I90N*^ (*Char4*) is further modified by the *Char9* locus on chromosome 10 which causes increased malaria-susceptibility due to the loss of pantetheinase activity (*Vnn3*) and its key metabolic product, cysteamine[17,43,44]. On the other hand, and despite carrying the *Pklr*^*I90N*^ mutation, AcB62 mice are susceptible to *P*. *chabaudi* infection, and show high parasitemia at the peak of infection with significant mortality[19]. Mapping studies in an informative F2 cross generated between AcB62 (*Pklr*^*I90N*^) and CBA/Pk (*Pklr*^*G338D*^) identified the major genetic modifier of Pklr-associated malaria resistance to be linked to chromosome 9 (*Char10*; LOD = 7.24) with additional effects linked to segregation of functionally distinct mutant *Pklr* alleles mapping to chromosome 3 (LOD = 3.7)[19,27].

A first aim of our study was to establish the effect of the *Char10* locus on the modulation of PK-deficiency associated resistance to blood-stage malaria. For this, we generated an incipient congenic line (Char10C) in which the *Char10* alleles of AcB62 were introduced onto genetic background of the CBA/Pk mouse strain by continuous marker-assisted backcrossing. With respect to reticulocytosis and response to malaria, we observed that the Char10C line recapitulates the phenotype of AcB62, displaying reduced blood reticulocytes at steady state and high parasitemia following *P*. *chabaudi* infection (Fig 1). This established that *Char10* is the major regulator of both phenotypes, with phenotypic expression being fully penetrant. Hence, comparative analyses in the CBA/Pk and Char10C strain pairs was used to study the effect of *Char10*.

We observed that, compared to the CBA/Pk parent, the Char10C mice display a reduction in anemia phenotypes associated with the *Pklr*^*G338D*^ mutation including decreased splenomegaly, diminished circulating reticulocytes, increased density of mature erythrocytes, increased hematocrit, as well as decreased iron overload in kidney and liver and decreased serum iron and transferrin-bound iron (Figs 2, 3 and 4). Several possibilities must be considered to explain the role of *Char10* in seemingly regulating penetrance and/or expressivity of the anemia phenotypes associated with Pklr-deficiency. First, *Char10* may directly influence the effect of loss of Pklr function on RBC metabolism including fragility. This appears unlikely, as Pklr-deficient erythrocytes have the same half-life in Char10C and in CBA/Pk mice (Fig 5A), and normal erythrocytes are cleared with similar efficiency by both mouse lines (Fig 5B and 5C). Second, it is possible that *Char10* affects a sensing mechanism of the host that detects signals of haemolytic anemia, a hypothesis we cannot formally exclude. Thirdly, *Char10* could regulate the extent of compensatory erythropoiesis in response to the same haemolytic insult caused by the *Pklr*^*G338D*^ mutation. This could manifest itself either through cell-autonomous mechanisms affecting the potential of individual erythoblast precursors to mature into erythrocytes, or though modulation of the total number of erythroblasts produced in the global response to haemolytic anemia. The data presented in Figs 6 and 7 argue against a cell-autonomous effect of *Char10* on intrinsic differentiation potential of erythroblasts *in vivo* and CFU-E colony formation *ex vivo*. This is clearly distinct from mutations that affect regulation of pro- or anti-apoptotic response in erythroblasts in response to EPO[45]. Finally, it is possible that *Char10* has pleiotropic effects on a combination of the above-mentioned host response pathways. Nevertheless, our results establish *Char10* as a critical regulator of erythropoietic response (compensatory erythropoiesis) to haemolytic anemia in pyruvate kinase deficiency.

Identifying the gene and protein underlying the *Char10* effect will be of considerable interest, possibly providing new targets for intervention in conditions such as haemolytic anemia. Currently, the *Char10* interval stands at >35Mb (size of the congenic segment fixed in Char10C mice) and contains >200 genes; Hence, it is not possible to discuss the implication of potential positional candidates in the phenotype. Because the minimal physical interval of the *Char10* locus remains large, we cannot formally exclude the possibility that *Char10* is a complex locus involving the combined effect of several tightly linked genes. It is also possible that such tightly linked genes may affect different physiological functions, including but not limited to erythropoiesis, and/or host immune functions. In addition, the Char10C line is not fully congenic, as it was backcrossed for only 4 generations before breeding to homozygosity. Hence, we cannot formally exclude the possibility that other portions of the genome derived from AcB62 remain in the Char10C incipient congenic strain. Nevertheless, it is worth mentioning that *Char10* maps to a portion of chromosome 9 that harbors a previously published quantitative trait locus designated *Splq4* and that regulates spleen weight in a large F2 cross between parental strains selected for large differences in body weight[46]. It is tempting to speculate that the same gene underlies *Char10* and *Splq4* effects. Irrespective of the molecular basis of the *Char10* effect, it will be interesting to determine whether or not *Char10* can modulate penetrance and expressivity of other gene mutations that are known to cause defects in erythrocytes function and that protect against malaria, including hemoglobin genes (sickle cell anemia, α/β thalassemias), ankyrin deficiency, G6PD-deficiency and others.

## Supporting information

S1 Table Blood cellular profile of CBA/Pk and Char10C. (XLSX)

S1 Fig Numbers of CFU-E progenitors in bone marrow (BM) of CBA/Pk, Char10C and C57BL/6 mice. Femurs of three male mice (one femur per mouse) were pooled for each strain. Erythropoietin titration was performed on CBA/Pk femurs. Histograms represent the mean of two technical replicates. (A) Erythropoietin titration. (B) Total number of CFU-E per femur at 10mU/mL of EPO. (C) Number of CFU-E per 4x10^4^ cells plated at 10mU/mL EPO.
(TIF)
