# Supplementary figures and images for: The mouse Char10 locus regulates severity of pyruvate kinase deficiency and susceptibility to malaria
Source: PLoS One. 2017 May 18;12(5):e0177818. doi: 10.1371/journal.pone.0177818 (PMC5436716; doi:10.1371/journal.pone.0177818)

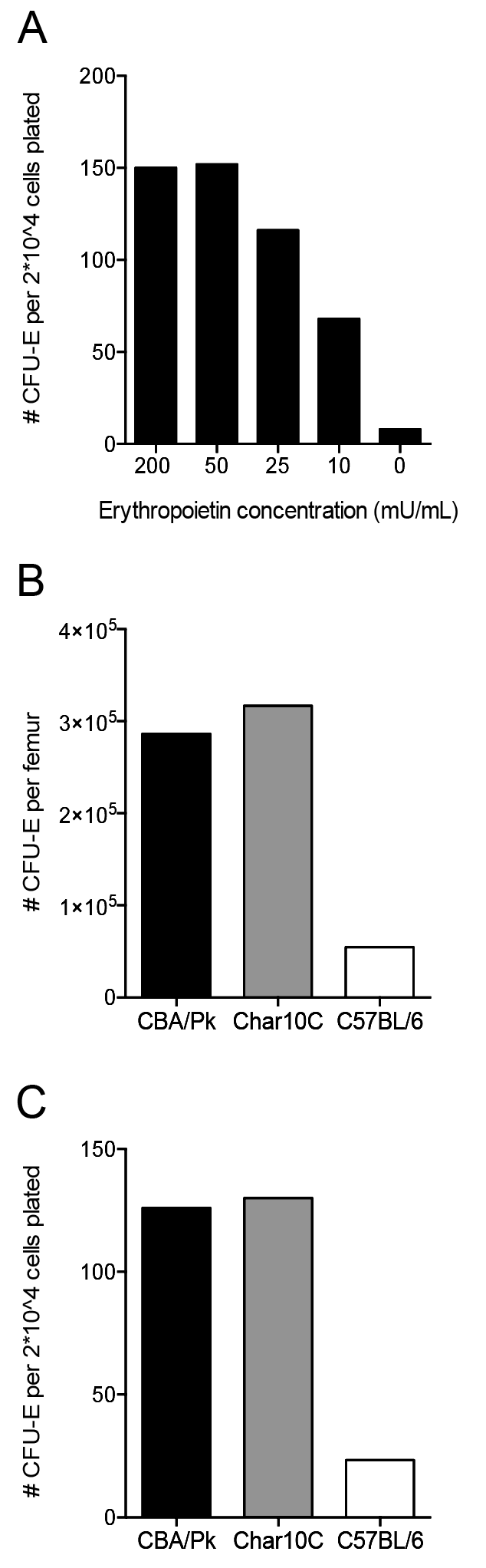

Supplement: S1 Fig — Femurs of three male mice (one femur per mouse) were pooled for each strain. Erythropoietin titration was performed on CBA/Pk femurs. Histograms represent the mean of two technical replicates. (A) Erythropoietin titration. (B) Total number of CFU-E per femur at 10mU/mL of EPO. (C) Number of CFU-E per 4x10^4^ cells plated at 10mU/mL EPO. (TIF) [file pone.0177818.s002.tif]
